# EHR4CR local workbench

**DOI:** 10.1186/2043-9113-5-S1-S12

**Published:** 2015-05-22

**Authors:** David Voets

**Affiliations:** 1 Custodix, Kortrijkse Steenweg 214, Bus 3, 9830 Sint-Martens-Latem, Belgium

## Characterisation

Tool, recruitment process, multi-centric clinical trials.

## Description

**Figure 1:**
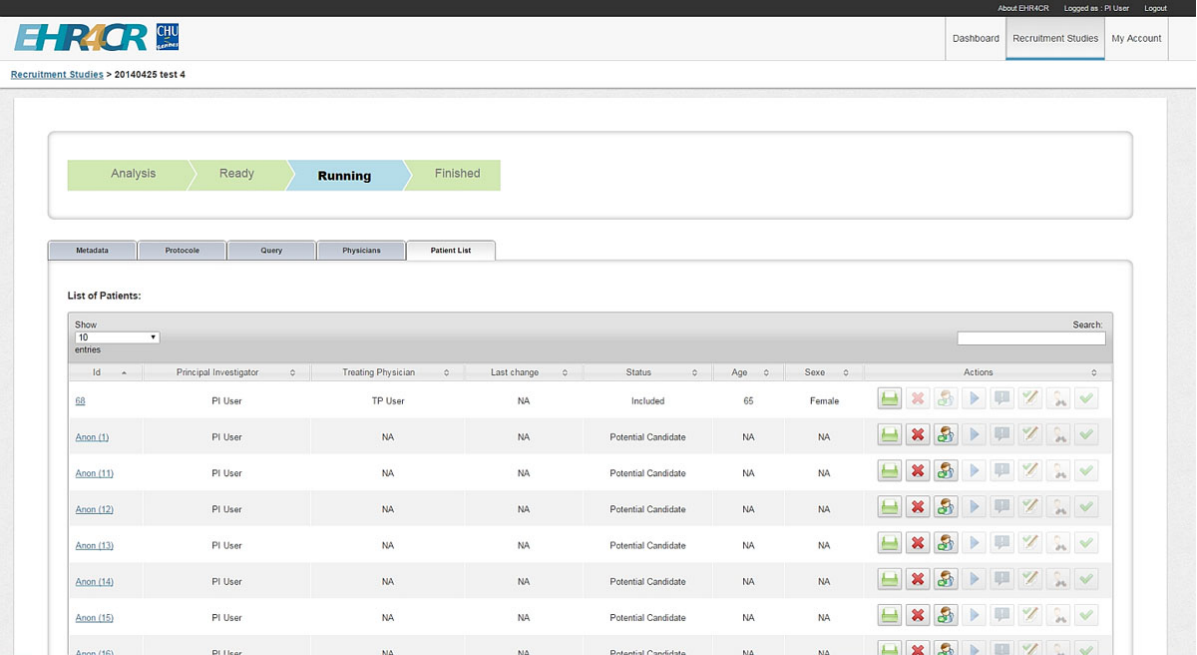
User interface of the local workbench indicating candidate patients for trial participation.

This tool supports administering and monitoring the recruitment process for multi-centric trials within a single clinical centre. It works together with the EHR4CR central workbench and allows receiving, accepting or rejecting invitations to participate in clinical trials (Figure 1). Local users can be assigned to clinical trials under a given role and engaged as such in different steps of the local subject identification and recruitment process. The tool also supports automatic fetching of a list of potential candidate patients for recruitment from a local clinical data warehouse based on formalized eligibility criteria included in the trial metadata. Once identified, potential candidate patients and their subsequent recruitment status can be managed and monitored (ranging from the status of potential candidate over confirmed candidate, patient in screening, patient has consented, etc.). Role-based re-identification of pseudonymised patient records avoids that the patient’s identity is exposed before the patient has been contacted by a treating physician and before the patient has agreed to participate in the recruitment process. The tool is a workflow-driven system that supports task assignment to end-users and triggering automatic periodic submissions of the clinical site’s recruitment numbers to the study sponsor or CRO.

## Status of development

Under evaluation by the EHR4CR pilot sites (November 2014).

## Users

Clinical sites (data relationship managers, (principal) investigators, study nurses, treating physicians).

## Links

http://www.ehr4cr.eu

